# TECHNOLOGY AND OLDER ADULTS: BEST PRACTICES FOR ENHANCING USABILITY, ENGAGEMENT, AND COMMUNICATION

**DOI:** 10.1093/geroni/igad104.1167

**Published:** 2023-12-21

**Authors:** Francesca Falzarano

**Affiliations:** Weill Cornell Medicine, New York City, New York, United States

## Abstract

The massive growth in population aging has coincided with a time of continued fast-paced innovations in technology. Technology can be leveraged to support health, well-being, and quality of life in older adults with health, mobility, and cognitive issues and is a means of maintaining social engagement in later life. However, for technology to achieve its intended purpose, older adults must have access to and recognize the value of such products. Despite the sheer size of the older adult population, the field of Information Communications Technology (ICT), has long overlooked the perspectives of older adults with regard the design of ICT applications. ICT systems that fail to consider the diverse capabilities and attitudes of older adult users are likely to alienate a significant, growing segment of the population and will likely be underutilized. This presentation will review best practices to enhance older adults’ usability and engagement with technology, with an emphasis on user-centered design principles as well as innovative tools facilitating virtual ICT support and training (e.g., TeamViewer software) to provide older adults who have limited to no technology proficiency with the skills needed to navigate its use. Key takeaways from this presentation will include a greater understanding of the importance of design principles to enhance usability and adoption of ICT systems among older adults; means of increasing access and adoption of ICT applications; and how user feedback can be leveraged to inform adaptions to enhance the efficacy and acceptability of technology applications, and ultimately foster psychosocial well-being in older adults.

